# Bat2Web: A Framework for Real-Time Classification of Bat Species Echolocation Signals Using Audio Sensor Data

**DOI:** 10.3390/s24092899

**Published:** 2024-05-01

**Authors:** Taslim Mahbub, Azadan Bhagwagar, Priyanka Chand, Imran Zualkernan, Jacky Judas, Dana Dghaym

**Affiliations:** 1 Department of Computer Science and Engineering, American University of Sharjah, Sharjah 26666, United Arab Emirates; b00074086@aus.edu (A.B.); g00069559@aus.edu (P.C.); izualkernan@aus.edu (I.Z.); ddghaym@aus.edu (D.D.); 2 Nature & Ecosystem Restoration, Soudah Development, Riyadh 13519, Saudi Arabia; jjudas@soudah.sa

**Keywords:** IoT, bioacoustics, bat echolocation analysis, bat species classification, LoRaWAN, machine learning, NVIDIA Jetson, Google Coral

## Abstract

Bats play a pivotal role in maintaining ecological balance, and studying their behaviors offers vital insights into environmental health and aids in conservation efforts. Determining the presence of various bat species in an environment is essential for many bat studies. Specialized audio sensors can be used to record bat echolocation calls that can then be used to identify bat species. However, the complexity of bat calls presents a significant challenge, necessitating expert analysis and extensive time for accurate interpretation. Recent advances in neural networks can help identify bat species automatically from their echolocation calls. Such neural networks can be integrated into a complete end-to-end system that leverages recent internet of things (IoT) technologies with long-range, low-powered communication protocols to implement automated acoustical monitoring. This paper presents the design and implementation of such a system that uses a tiny neural network for interpreting sensor data derived from bat echolocation signals. A highly compact convolutional neural network (CNN) model was developed that demonstrated excellent performance in bat species identification, achieving an F1-score of 0.9578 and an accuracy rate of 97.5%. The neural network was deployed, and its performance was evaluated on various alternative edge devices, including the NVIDIA Jetson Nano and Google Coral.

## 1. Introduction

The advent of compact and cost-effective acoustic sensors has revolutionized ecological research, particularly in the monitoring and analysis of wildlife and their natural habitats. This method has gained prominence for its passive yet insightful data collection [1]. Such data collection methods have been used for bats, whose complex echolocation system exhibits significant variability influenced by numerous factors [2]. Bats navigate and perceive their surroundings through the echoes returned from their environment by utilizing high-frequency pulses ranging from 9 kHz to 200 kHz, which is well beyond human hearing [3]. As nocturnal creatures, these echolocation calls serve as a crucial tool for researchers to study various aspects of bat populations. To this end, ultrasound microphones like the Pettersson DX1000, capable of capturing frequencies from 5 to 235 kHz, are employed by researchers. While signals in the time domain offer limited insights, frequency-based analyses of the spectrograms reveal distinct call shapes unique to each bat species [2,4]. However, variations within a species are common, with factors like habitat, wing morphology, and recording timing influencing call characteristics. Moreover, bats emit various sounds for behaviors like mating, expressing distress, or defending territory [2]. This complexity introduces inter-observer variability in the study of bat calls, emphasizing that accurate analysis demands significant expertise and time.

**Figure 1:**
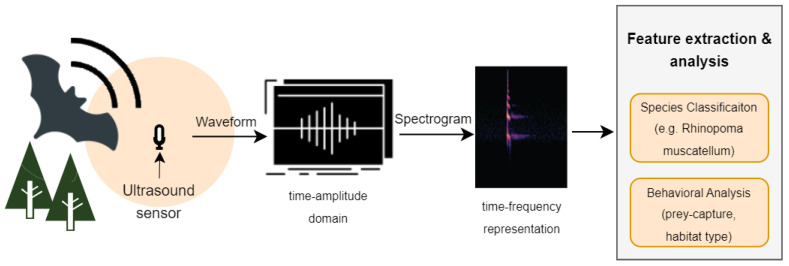
Recording and processing of a bat’s acoustic signal. The computed spectrogram (frequency-domain representation) is processed by a deep convolutional neural network, which extracts features pivotal for a spectrum of analysis.

The unique signatures within the echolocation calls of a particular bat species offer an innovative avenue for species identification. Utilizing neural networks (NN) trained to discern species based on these acoustic signals presents a promising alternative to manual monitoring, potentially circumventing its labor-intensive nature. The integration of artificial intelligence (AI) with the internet of things (IoT), commonly referred to as AIoT, stands at the forefront of this technological approach. Such systems harness advanced AI methodologies, particularly deep learning, to facilitate the remote classification of bat species. The ultrasound sensors employed in our study capture audio data rich in insights into bat species. Utilizing supervised learning algorithms, we can extract features from the recorded data to classify bat species via their echolocation calls. The workflow for our analysis is shown in Figure 1, demonstrating how signals from the time domain are converted into the frequency domain, which is essential for extracting features with a CNN model. These features are crucial for automated analysis, with this paper focusing specifically on species classification. This task is notably difficult without the aid of automated systems [1].

The application of machine learning (ML) models requires a significant amount of meticulously labeled data to establish an effective monitoring system. Our approach leverages expert knowledge to label a subset of bat data collected from the wild, focusing on the identification of species by analyzing frequency patterns within audio segments. Building on this annotated dataset, we use supervised learning methods to develop a neural network (NN) model for the classification of bat species, which is subsequently integrated into IoT edge devices for deployment. Our research used echolocation call recordings of bats from the Hajar Mountains in the UAE, documented by Emirates Nature (WWF) [5]. The data collection process entailed precise identification of bats through morphological measurements, capture via mist nets, affixing bioluminescent markers for species identification, and subsequent release. The Pettersson D1000X ultrasound detector was utilized to record calls from eight distinct bat species: Rhinopoma muscatellum, Taphozous perforatus, Pipistrellus kuhli, Rhyneptesicus nasutus, Eptesicus bottae, Rousettus aegyptius, Myotis emarginatus, and Asellia tridens.

The primary contributions of this study are as follows: Firstly, we have crafted a supervised neural network algorithm tailored for the real-time monitoring of bat species. This innovative aspect of our research introduces a custom-designed, lightweight convolutional neural network (CNN) model optimized for deployment across a spectrum of edge devices, demonstrating its versatility and efficiency. We have bench-marked the performance of this small model on a variety of hardware-edge devices. Finally, we have developed a detection-to-database transmission pipeline, facilitating an efficient end-to-end monitoring system. To our knowledge, this is the first study to openly provide the complete source code for a bat detection system to the community. This move aims to encourage collaboration and speed up advancements in bio-acoustic monitoring. With few open-source projects available in this field [6], our contribution offers a practical alternative to commercial options like Sonobat [7] or Kaleidoscope [8], making advanced research tools more accessible to researchers.

The remainder of the paper is structured as follows: Section 2 discusses existing literature and foundational knowledge pertinent to our study. Section 3 offers an in-depth examination of our proposed system, detailing the deep learning models employed, the evaluation metrics used, and the system-level details of the architecture. Following this, Section 4 presents a thorough analysis of our findings from the supervised approaches to studying bat echolocation calls. We conclude our paper in Section 5.

## 2. Background and Related Work

### 2.1. IoT Edge Devices

Integrating machine-learning capabilities into edge devices presents many challenges and requires careful consideration of many decision criteria [9]. Such architectures are particularly valuable in scenarios where the latency of data transmission and bandwidth constraints are critical. The selection of an appropriate edge device is highly dependent on the specific requirements of the application in question, including both the context and computational demands [10]. A commonly preferred option is the use of general-purpose microcomputers like the Raspberry Pi (RPI), which have been deployed in diverse applications ranging from IoT agricultural systems for disease monitoring in strawberry farming [11] to more traditional computing tasks [12,13].

In addition to general-purpose devices, specialized edge devices are designed explicitly for deep learning applications [10,14]. Such devices include the Nvidia Jetson Nano [15] and the Google Coral Accelerator [16], each with its own set of performance trade-offs. For example, Antonini et al. [17] conducted a comparison and reported that the Google Coral demonstrated significantly faster inference times across various CNN models, including those for audio processing, in contrast to the Nvidia Jetson Nano. Furthermore, it was observed that the Jetson Nano’s consumption of power was considerably higher when utilizing the TensorFlow-GPU framework as opposed to TensorFlow-RT, despite similar inference times. In a related study, Silveira et al. [18] benchmarked the Nvidia Jetson Nano against the RPI 3B+, employing the simultaneous localization and mapping (SLAM) algorithm. Their findings highlighted the nano’s superior performance, achieving 12.6 frames per second (FPS) and 12.1 FPS across two datasets, in stark contrast to the RPi 3B+’s 4.4 FPS and 3.6 FPS, respectively.

### 2.2. IoT Network Architectures

An IoT architecture, which processes data at the edge, significantly reduces network bandwidth requirements, making low-power wide-area network (LPWAN) technologies a suitable choice for various AIoT applications [19,20]. Among these technologies, LoRaWAN emerges as a good option due to its extended battery life and efficient power consumption, which is critical for remote applications like bat monitoring [10,21]. Although network alternatives like NB-IoT may offer better quality of service (QoS), LoRaWAN’s superior battery performance—up to ten times longer than NB-IoT—and the ability to drastically reduce data transmission make it ideal for deployments in areas lacking reliable internet access or where devices are solar-powered [21].

Comparative studies, such as those by Lalle et al. [22], highlight LoRaWAN’s advantages over NB-IoT and SigFox, including better battery life and lower latency, further underscoring its suitability for IoT-based monitoring tasks. Practical implementations in diverse environments have demonstrated LoRaWAN’s impressive range, from up to 9 km in urban settings to 47 km in rural landscapes [23]. This capability has been leveraged in various real-time IoT monitoring systems, from smart irrigation and agriculture to wildlife tracking, showcasing LoRaWAN’s broad applicability and efficiency in reducing power consumption by up to 50% compared to baseline networks [24,25,26,27]. To sum up, while there are several network technologies available for AIoT applications, LoRaWAN stands out for bat monitoring and similar applications in the wild due to its low power consumption and effective latency management, making it a preferred choice for enhancing the sustainability and reach of IoT deployments.

### 2.3. The Internet of Audio Things

The Internet of Audio Things (IoAuT) [28] represents a specialized IoT system centered around audio data collected from diverse environments. Unlike the internet of musical things (IoMuT), which caters exclusively to musical interactions with human stakeholders, IoAuT encompasses a broader range of applications, including environmental and wildlife monitoring. The distinction between IoAuT and IoMuT extends to the specific challenges each faces, such as latency, audio quality, and data pre-processing, which differ markedly due to their unique operational contexts [28]. A key application of IoAuT is ecosystem monitoring, often referred to as “eco-acoustics”, which plays a critical role in assessing the health and diversity of natural habitats [28]. Research [29] has shown that audio data, especially from microphone arrays, serves as an effective tool for estimating the density and identifying the species of animals, including birds and bats. However, bat species recognition presents a significant challenge due to the complexity of their vocalizations [29]. Additionally, IoAuT technologies enable acoustic localization, pinpointing the positions of animals based on their sounds, which is invaluable for tracking and studying wildlife behavior [30].

### 2.4. Classification Based on Audio Data

Deep learning has revolutionized audio-based classification, outperforming traditional methodologies with significant advancements [31]. The process typically involves converting audio data into spectrograms, which serve as visual representations, which are then analyzed by supervised learning algorithms, particularly CNNs, to identify unique features. In the medical field, Kutsumi et al. [32] utilized a CNN to analyze bowel sounds captured by smartphone microphones as a non-invasive measure of gastrointestinal health. Similarly, Peruzzi et al. [33] explored the use of acoustics for diagnosing bruxism-related disorders, and Tariq et al. [34] leveraged audio data from lung and heart sounds for the classification of various medical conditions, contributing to the advancement of early disease diagnostics across multiple health domains. Additionally, Henry et al. [35] focused on enhancing auditory systems, employing Mel spectrogram features from speech to synthesize improved auditory outputs for cochlear implant users. Beyond healthcare, Di Maggio [36] applied knowledge transfer techniques within the audio domain to train a CNN model for the diagnosis of industrial bearing faults with a remarkable accuracy of 99.07%. Similarly, Jung et al. [37] utilized 2D sound spectrograms in conjunction with CNNs to detect rotor failures, achieving a validation accuracy exceeding 99%. Earlier, Tran et al. [38] employed Mel-frequency cepstral coefficients (MFCC) features from drill sounds to develop a classifier capable of categorizing drill noise into three distinct categories for automated fault detection.

Within the realm of acoustic environmental research, AI methodologies have been effectively leveraged in a diverse array of studies. These include the detection of orca whales [39] and dolphins [40], the classification of fish [41], owl species [42], and bird songs [43]. Additionally, some research has integrated deep learning with traditional machine learning (ML) approaches for enhanced classification of anurans, birds [44], and robust bird classification [45]. Beyond supervised learning, unsupervised learning techniques have been employed to analyze marine audio data, clustering distinct sounds such as those from fish, shrimp, and ships, using methods like long-term spectrogram (LTS) and periodicity-coded non-negative matrix factorization (PC-NMF) for effective data visualization and separation [46]. Potamitis [47] demonstrated the efficacy of unsupervised learning in clustering bird audio calls from extensive field recordings, achieving an impressive F1-score of 0.88 in bird call detection with a Random Forest algorithm, showcasing the broad applicability and potential of deep learning in audio classification across diverse ecological studies.

Significant progress has been made in the field of bat species analysis via deep learning. Aodha et al. [4] employed a CNN model to detect bat calls within audio streams, drawing upon data from various European regions, and achieved an impressive F1-score of 0.8548. Earlier, Hughes et al. [48] utilized discriminant functional analysis (DFA) on manually grouped audio calls of Thai bat species based on frequency ranges, with classification accuracies of between 70.4% and 96.7%. The application of DFA extended to field sampling enhancements [49] and canonical discriminant analysis for analyzing echolocation call structures [50]. Tabak et al. [51] employed the frequency-over-time data derived from zero-crossing (ZC) echolocation signals to train a CNN, successfully classifying 10 species of bats with a testing accuracy of 90%. This performance markedly surpassed that of two commercial software solutions, Kaleidoscope Pro and BCID. Similarly, Paumen et al. [52] harnessed MFCC as features to train a CNN, attaining a classification accuracy of 96% for bat species. Alipek et al. [6] introduced a novel supervised model leveraging compressed spectrograms derived from unsupervised UMAP clustering for species and genus classification, with F1-scores ranging from 92.3% to 99.7% and 94.6% to 99.4%, respectively. However, unsupervised methods like k-means clustering fell short in comparison [53]. This review underscores a critical gap: the need for an integrated end-to-end monitoring pipeline incorporating lightweight supervised classification models for efficient bat species detection.

## 3. Methodology

In the following section, we describe the architecture of the proposed system, encompassing the system device and protocols, the data pre-processing pipeline, and the implementation of the supervised model. Our source code is publicly available on GitHub at https://github.com/Taslim-M/Bat2Web (accessed on 5 February 2024).

### 3.1. System Architecture

**Figure 2:**
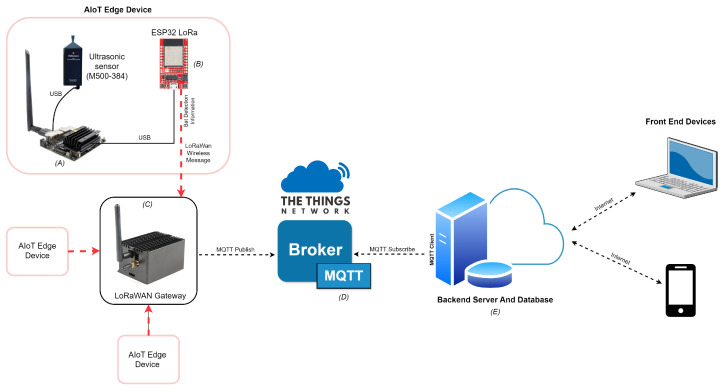
IoT system architecture diagram. (**A**) illustrates the edge device connected to the ESP-32 LoRaWAN device in (**B**). The detection data is relayed to the LoRaWAN gateway depicted in (**C**) and subsequently forwarded to The Things Network (TTN), which is highlighted in (**D**). Finally, TTN sends the message to the application server (**E**) using the MQTT broker.

Figure 2 shows the detailed architecture of our proposed bat species monitoring system, employing IoT technology. This system is characterized by the integration of edge devices, each outfitted with a microphone and an embedded edge device for on-site deployment of a CNN model. Specifically, the system utilizes a high-fidelity 16-bit, 384 kHz analog-to-digital (A/D) Pettersson M500-384 USB Ultrasound microphone for audio signal acquisition.

The edge devices establish a connection with the microphone via USB 3.0 interfaces. These devices are responsible for capturing audio signals, processing them through the CNN to classify bat species, and subsequently transmitting the processed data to a cloud-based server. This transmission is facilitated through a LoRaWAN network, connecting to the server via a LoRaWAN gateway. The data relayed includes crucial information such as the identified bat species, the corresponding time stamp, and the geographical location of the detected call. A detailed specification of the ultrasound sensor used in the system is presented in Table 1.

### 3.2. System Server and Front End View

The back-end architecture, comprising both the server and database, provides an advanced interface designed for researchers to efficiently access, analyze, and interact with the echolocation data recordings. The application server, functioning as the primary node, handles HTTP requests, orchestrating the retrieval, processing, and presentation of data alongside the generation of HTML content for end-user interaction. This system encapsulates several critical operations, including a dynamic filtering mechanism that accommodates precise data retrieval based on user-specified criteria. Furthermore, the development of a comprehensive dashboard is undertaken to present aggregated data through visual graphs, offering an intuitive summary of key findings for researchers. A pivotal feature includes the integration of geo-spatial visualization, enabling the mapping of bat territories for enhanced geographical analysis. This functionality leverages the Google Maps API (3.44.2), selected for its extensive adoption and its capability to render diverse terrains via Google Earth, thereby facilitating a versatile platform for ecological study. The web application undergoes rigorous unit testing to ascertain functionality and reliability, with a steadfast commitment to adhering to W3C standards (WCAG 2.0). This ensures the application’s compliance with global web protocols, optimizing interoperability and the user experience across diverse computing environments.

### 3.3. System Network Connection

For this application, a traditional network protocol for communications, such as Wi-Fi or 3G, would not be suitable as the edge devices are deployed in remote areas where coverage may not be available. While Wi-Fi can be used for transmitting data between a fixed central gateway and the application server, the edge devices are not fixed. Researchers are able to deploy them flexibly in any location in a given habitat, such as a rainforest. No static network infrastructure can be relied upon due to this flexible deployment ability. Furthermore, power consumption considerations for the network component are critical, as the edge device will rely on low-power charging methods like solar panels. Since the edge devices will be remote and will not be exposed to sunlight at all times, a conservative approach must be taken to power consumption planning. Hence, a network protocol was required that allowed for long-range communications and utilized relatively less power.

From the literature review, it was concluded that LoRaWAN was the most apt protocol for our particular use case. Firstly, LoRaWAN possesses a suitable range in the order of 10s of kilometers without having to rely on existing infrastructure like Wi-Fi. This helps ensure that the edge devices can flexibly be deployed over a given bat habitat, allowing researchers to explore and study a wide area of the region. Secondly, when compared to other candidates for such a protocol, like NB-IoT, LoRaWAN has been shown to demonstrate significantly lower power consumption. Given that the edge device must be a small and inaccessible component that already utilizes some of its power to collect audio data and process the NN model, it is essential to be as frugal as possible with the network communication power requirements. The specifications for the utilized LoRa Gateway (ESP32) are presented in Table 2.

The review also indicated that there are some issues with LoRaWAN’s choice. For example, other network protocols, like NB-IoT, have demonstrated higher QoS during their transmissions. However, this was not considered to be a critical issue because the nature of our particular payload is not highly sensitive. For a given bat detection event, there are likely to be many repeated detections of the same bat that are transmitted to the server. Even if some of these transmissions are dropped by the network, overall, the detection will most likely still be captured in our system. The volume of data being transmitted implies that a few missed transmissions due to low QoS will not massively impact the system’s performance, unlike some other more sensitive applications. Nevertheless, this is a disadvantage of the choice of LoRaWAN that must be considered.

The outline of the specific LoRaWAN setup that is used for implementing the proposed system is as follows: The edge device, labeled Figure 2A, is directly connected to the ESP-32 LoRa Gateway (setup as a LoRaWAN device), labeled Figure 2B, to enable LoRaWAN messages to be transmitted from the edge device in the event of a species detection. This is done via the CH340C USB-serial bridge port connected via a USB cable to the edge device. The edge device uses the serial Python library to open a serial connection to the USB port to which this cable is connected. Whenever a bat detection is registered, a comma-separated string is written to this serial connection, consisting of the species code, latitude, and longitude. The ESP-32 was programmed using Arduino and the Arduino-LMIC library. This Arduino script features an endless loop that, if a payload is detected on the serial connection, transmits the same string to the LoRaWAN Gateway, labeled Figure 2C. This is done by registering the ESP-32 as an application on The Things Network web portal.

The LoRa Raspberry Pi Gateway serves as the middleman during the communications, as it will receive the messages from the edge device over the LoRaWAN protocol and forward them to The Things Network (TTN) broker, labeled Figure 2D. This gateway was configured to follow the relevant LoRa channel for our region (EU 863-870) and was registered in The Things Network web portal. The specifications for the LoRaWan Gateway are presented in Table 3.

The TTN uses an MQTT broker, which forwards the packets to the application server, labeled Figure 2E, over the internet. The application server is an MQTT subscriber and receives the messages published by the LoRaWAN RPi gateway. Specifically, it is a subscriber to the topic “esp32-device-bat-proj-/devices/second-esp32-edge-node/up”. The original string is now parsed using JavaScript string operations and converted to an object. This object is then saved to the MongoDB database.

### 3.4. Edge Device Selection

In our study, we conducted a comprehensive comparison of edge devices equipped with TPU, GPU, and CPU capabilities to identify the optimal hardware for deploying a neural network with reduced inference response time suitable for real-time applications. The evaluation encompassed a range of widely used devices, including the Raspberry Pi (RPi) 3B+ and 400 (CPU), NVIDIA Jetson Nano (GPU), and Google Coral (TPU). TPUs, designed with a grid of simplified ALUs and leveraging a pipelining effect across an nxn matrix, offer low memory requirements and reduced power consumption due to minimal memory access [55]. GPUs, utilizing tensor cores, excel at performing large, parallel matrix operations but typically involve longer training and inference times due to higher calculation precision. CPUs, with their multiple cores and high processor frequencies, provide an alternative by facilitating inference through interconnected compute nodes, with their effectiveness slightly constrained by memory bandwidth limitations. The selected edge devices were rigorously assessed based on critical metrics pertinent to IoT deployments: power consumption, latency during model inference, and CPU utilization. These factors are crucial in determining the suitability of hardware for IoT applications, influencing both operational efficiency and the feasibility of real-time processing [9,10].

### 3.5. Data Preprocessing

In our methodology, supervised learning tasks were conducted using an image-based representation of the raw audio recordings. Each audio (WAV) file was segmented into 3-s intervals and subsequently converted into spectrograms utilizing the Librosa Python library. We experimented with three types of feature representations as inputs for the machine learning models: short-term Fourier transform (STFT), Mel-scaled filter banks (MSFB), and Mel-frequency cepstral coefficients (MFCC). In terms of the order of processing these features, STFT is calculated first, followed by applying Mel filters to create an MSFB, and then optionally doing a discrete cosine transform (DCT) to create an MFCC plot. DCT is often employed to get rid of correlations among Mel filter bank coefficients in the MSFB representation. Among these, the MSFB representation yielded the most favorable results, as detailed in our prior research [56]. Before processing, the audio files were downsampled to a sampling rate of 44.1 kHz, shifting the bat call frequency pattern without changing their shape structure. This downsampling reduces the size of the resulting spectrogram, effectively decreasing the number of parameters required by the model. As an example calculation regarding the CNN structure described in Section 3.6, using a sampling frequency of 342 kHz to produce the spectrograms results in the model’s parameters increasing to 432k. This is more than double the 204k parameters obtained when spectrograms are generated at 44.1 kHz. The data processing steps employed are as follows:

1. Audio WAV files were imported utilizing the Librosa library, adhering to a sampling rate of 44,100 Hz.
2. Each audio file was segmented into fixed lengths of 3 s each; this threshold was determined to be a safe margin with a probability of approximately 90%, where at least one bat call could be seen.
3. Upon import of the 3 s audio segment, spectrograms were generated through short-time Fourier transform (STFT) employing a Hann window of 25 ms with a hop length of 10 ms, resulting in a 15 ms overlap.
4. Subsequent to STFT, Mel-scaled filter banks (MSFB) were constructed from the power spectrum using 128 mel filters, followed by logarithmic transformation of the filter bank outputs.
5. Finally, a discrete cosine transform (DCT) was executed, with the preservation of 20 MFCC coefficients for subsequent MFCC analysis.

**Figure 3:**
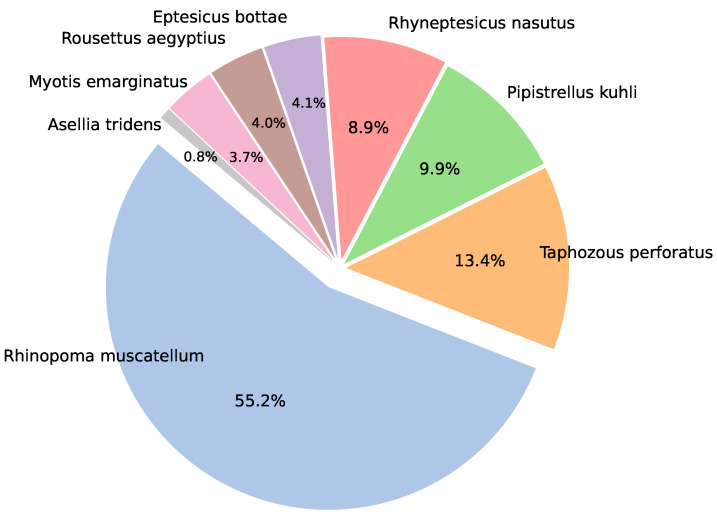
Distribution of data across the 8 bat species. A total of 3018 audio segments were converted to spectrograms to experiment with the CNN model.

The distribution of the dataset, comprising 3-s audio samples, is shown in Figure 3, highlighting a significant imbalance across the classes. Specifically, the dataset reveals a pronounced disparity, where the class represented by Rhinopoma muscatellum possesses approximately 60 times the amount of recorded audio compared to that of the least represented class, Assellia tridens. Training with imbalanced data can sometimes impact the ML model’s performance. Hence, we prepare an oversampling method for the minority samples in the training pipeline using SMOTE [57].

**Figure 4:**
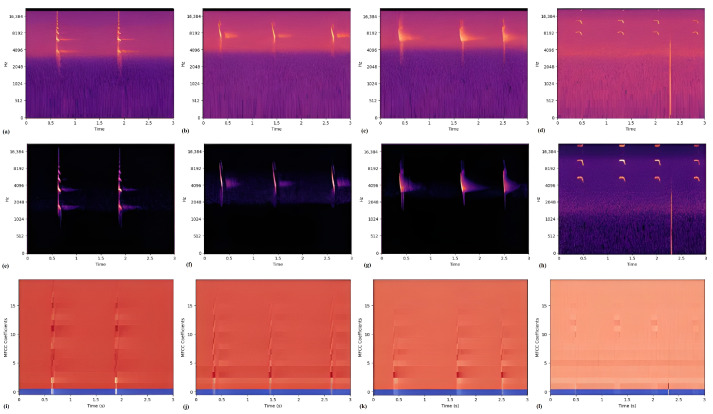
Short-time Fourier transform (STFT), Mel-scaled filter bank (MSFB), and Mel-frequency cepstral coefficients (MFCC) samples, respectively. (**a**,**e**,**i**) Pipistrellus kuhli. (**b**,**f**,**j**) Rhyneptesicus nasutus. (**c**,**g**,**k**) Taphozous perforatus. (**d**,**h**,**l**) Asellia tridens. The amplitude (energy) of the signals is represented with the varying color intensity.

Figure 4 presents sample images across three distinct representations for four bat species, illustrating both the similarities and unique frequency band characteristics captured in each. Among these, the MSFB representation notably accentuates the specific patterns of bat calls, making it the most distinctive. For scenarios necessitating data transmission, the MFCC representation emerges as a viable option due to its reduced storage demands. Nevertheless, the MSFB representation has demonstrated superior performance in conjunction with CNN models. An initial approach to noise reduction involved manual curation, specifically removing images that were evidently devoid of data. Although automated noise removal tools were initially considered [4], they proved excessively stringent for our purposes, leading to the undesirable exclusion of potentially informative data. Consequently, a method of visual inspection was adopted for noise management in this phase. For future endeavors, particularly those involving extensive datasets, the utilization of open-source software like BatDetect (v1.0.8) [4] is advocated to streamline this process.

### 3.6. CNN Model for Species Classification

We created a supervised convolutional neural network (CNN) model specifically designed for the classification of bat species on edge devices, leveraging the Mel-scaled filter bank (MSFB) representation for audio analysis as established in our preceding work [56]. Our preliminary investigations showed an improvement in model accuracy when utilizing MSFB features—achieving an increase of 2.5% over STFT features and 3.9% over MFCC features. This improvement is corroborated by the distinct visibility of bat classes when analyzed with MSFB features, as illustrated in Figure 4. The diminished resolution of higher frequencies in MFCC features affects the accuracy of representing echolocation calls within the higher frequency range. This limitation contributes to the inferior performance during CNN training. Given that a considerable portion of echolocation calls occur at the higher end of the frequency spectrum, relying on MFCC features could lead to a less effective differentiation of bat calls, particularly at higher sampling rates.

With a focus on edge deployment, the model was engineered to maintain a small size. Built using the TensorFlow framework, the architecture of this CNN, presented in Table 4, incorporates convolutional layers for initial feature extraction from the input spectrograms, followed by dense layers responsible for the classification task. Batch normalization was integrated to enhance model stability and performance, standardizing the output from each layer. Pooling layers contribute to downsampling, aiding in feature generalization, while spatial dropout and traditional dropout techniques were implemented to mitigate overfitting by intermittently deactivating certain filters and node outputs. The model culminates in the application of a SoftMax activation function, facilitating the probabilistic classification of bat species. During the development of the model, attention was dedicated to optimizing the hyperparameters. This optimization process included varying the size of convolutional filters (choosing between 3, 5, or 7), the number of filters (ranging from 56 to 512), the number of nodes within dense layers (spanning 48 to 512), the count of dense layer groups (from 1 to 3), and adjusting dropout rates (0 to 0.1 for spatial dropout and 0.1 to 0.4 for regular dropout) to prevent overfitting. To achieve efficient and systematic hyperparameter tuning, the Hyperband algorithm, implemented through the keras-tuner tool [58], was utilized.

For the training phase, the CNN was fed with labeled spectrograms, utilizing an NVIDIA Quadro RTX. The model was trained with a batch size of 8 and using a learning rate of 0.003, employing the Adam optimizer. This CNN model is particularly suited for edge deployment, given that its total parameter count is approximately 200k. To further refine the model for edge device compatibility, we converted it into more compact formats, including TensorFlow Lite and TensorRT. These conversions were instrumental in reducing both the parameter count and the overall size of the CNN model. In addition to evaluating the model’s top 1% accuracy, the F1-score was also employed as a performance metric, considering the imbalanced nature of our dataset. The F1-score was calculated using the formula provided in Equation (1).

(1) $$F1\mathrm{-Score}=2\times \frac{\mathrm{Precision}\times \mathrm{Recall}}{\mathrm{Precision}+\mathrm{Recall}}$$

To provide a thorough evaluation of our CNN model’s performance, we present the confusion matrix alongside precision, recall, and AUROC (area under the receiver operating characteristic) scores for each class. Recognizing the dataset’s imbalance, as depicted in Figure 3, we employed a stratified sampling approach to construct a test dataset that constitutes 20% of the total data. Table 5 shows the quantity of data available in each training, validation, and testing set.

In the training phase, we allocated 20% of the data from the training folds to serve as the validation set. To guarantee the reproducibility of our findings, we used a consistent seed to divide the datasets for training and testing purposes. The test data were maintained as unseen datasets across all evaluations to ensure the integrity of our results. The purpose of the validation set was to track the progress of the training and assist in fine-tuning the hyperparameters. We implemented the SMOTE (neighbors = 5, equal classes) as an oversampling strategy, affecting only the quantity of training samples. Specifically, this technique was used to augment the size of smaller classes to equal the sample count of the largest class, which contained 1199 samples, thereby equalizing the representation of each class in the training dataset.

#### Semi-Supervised Model

**Figure 5:**
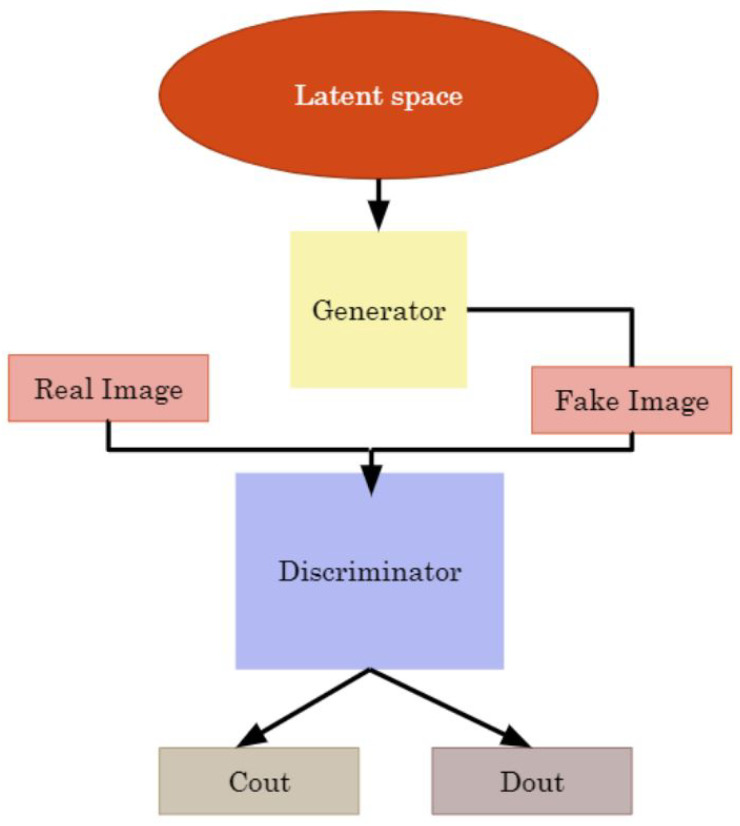
SGAN Architecture.

Given the complexity and time-intensive nature of labeling echolocation data, necessitating extensive domain knowledge, recent advancements have highlighted the efficacy of semi-supervised learning techniques like fixmatch and mean teacher in capitalizing on unlabeled data within audio classification tasks [59]. To obviate the need for fully labeled data, we further explored the use of a semi-supervised generative adversarial network (SGAN), previously validated for its robustness in processing physical sensor data [60]. SGAN innovatively combines a generator and discriminator in a unified framework, wherein the generator’s primary role is to synthesize deceptive, unlabeled images from latent space noise, thereby enhancing the discriminator’s accuracy, as depicted in Figure 5.

The discriminator, integrated within our proposed supervised CNN model (outlined in Table 4), operates with dual outputs, functioning as two distinct models with shared weights. The first output, C_out, caters to the supervised aspect, differentiating among the 8 bat species (with 8 classification outputs) through Softmax activation and sparse cross-entropy loss. The second output, D_out, addresses the unsupervised segment, discerning between fake and real images, utilizing a specialized activation function alongside binary cross-entropy loss. This hybrid approach, merging labeled and unlabeled data, substantially amplifies the discriminator’s classification precision beyond the capabilities of models trained solely on labeled datasets. The generator’s only objective is to use random noise in the latent space and generate fake, unlabeled images to improve the discriminator’s performance. The generator used in our experiment is presented in Table 6.

The generator’s latent space dimensionality in our experiments was configured to 100, utilizing a standard normal distribution to generate latent vectors. The generator transformed these vectors into synthetic images, termed ‘fake images’, which constituted a defined proportion of the training dataset for the discriminator. To assess the classification performance of the SGAN model (C_out), we employed the same seed and data distribution ratio for splitting the testing dataset in the supervised algorithm (see Table 5), ensuring a consistent benchmark for comparison. In each epoch, a random number of samples is selected from the training data to represent the real data in the supervised samples. The selection depends on the proportion of real data defined in the experiment. Since SGAN training was aimed at assessing performance under data-limited conditions, we did not apply SMOTE to the training data. In each training epoch, the discriminator’s weights are trained twice—one for real and another for fake images—whereas the generator is trained once.

## 4. Results and Discussion

In this section, we conduct a comprehensive analysis of our findings, showing the performance of the supervised deep learning model for species classification and an evaluation of the overall output of the integrated end-to-end bat monitoring system.

### 4.1. Species Classification Using Supervised Learning

**Figure 6:**
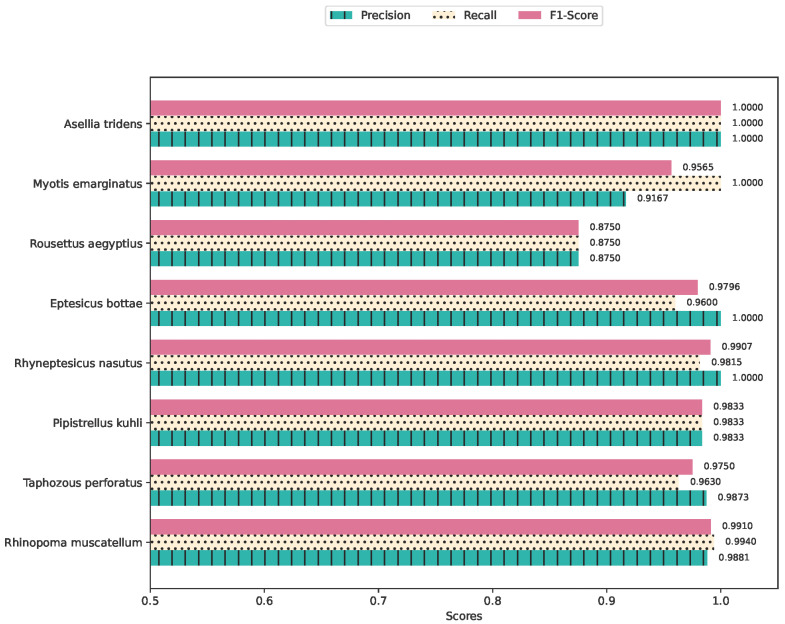
Average Precision, Recall, and F1-Score Values Per Species.

**Figure 7:**
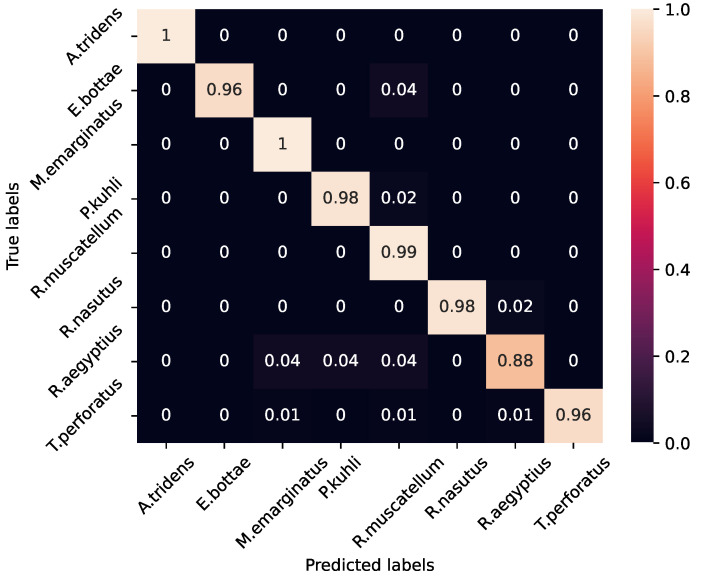
Normalized confusion matrix for the best performing model. The performance of the minor classes suggests that the CNN effectively discriminates features from the limited data of echolocation signals.

The convolutional neural network (CNN) model, tailored for the classification of labeled bat species, showed good performance, achieving an average test accuracy of 97.5% with a standard deviation of 0.9% and a mean F1-score of 0.9578, with a standard deviation of 0.02 using the MSFB features. These metrics, quantitatively denoted with their respective standard deviations, underscore the model’s consistent reliability and accuracy in classification tasks. Given the dataset’s imbalanced nature, a detailed classwise evaluation is imperative to gauge the model’s capacity for feature extraction and differentiation across varied species more accurately. The precision, recall, and F1-Scores for each class shown in Figure 6 show uniformly high values across all species, with the exception of Rousettus aegyptius. The confusion matrix depicted in Figure 7 shows the details of errors. During model optimization, training and validation loss curves showed no overfitting.

**Figure 8:**
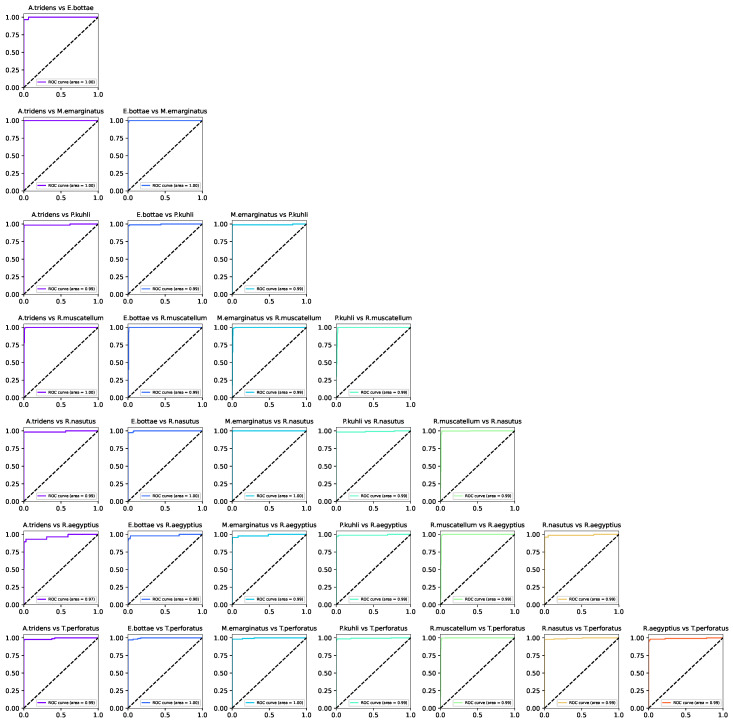
Pairwise ROC curves with the AUC score for each curve.

A pairwise ROC analysis was also conducted, with the outcomes shown in Figure 8. This analysis reaffirms our initial observations, showcasing area under the curve (AUC) scores spanning from an impressive 0.97 to an optimal 1.0, predominantly achieving the latter. These findings underscore the proposed model’s superior or comparable efficacy against contemporary alternatives while highlighting its advantage as a significantly more efficient neural network in terms of computational resource requirements. For misclassifications, particularly those concerning the R. aegyptius class, it becomes evident that environmental noise significantly contributes to prediction inaccuracies. Additionally, the variation within species—attributed to differences in foraging behavior and habitat conditions (open versus closed environments)—emerges as a factor in some misclassifications. This suggests that bat calls for a given species may exhibit noticeable differences in varying ecological contexts. Enriching our dataset to encompass a broader spectrum of echolocation call variations within species is posited as a strategy to enhance the classifier’s accuracy.

**Figure 9:**
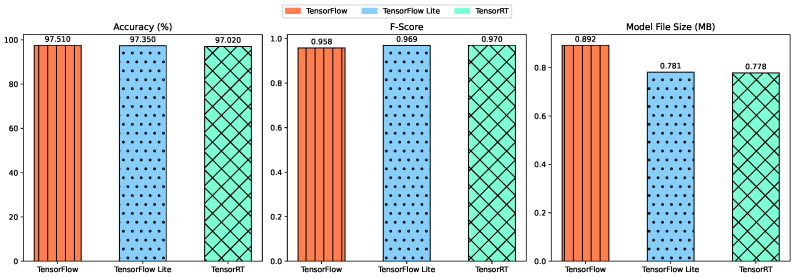
Performance and Size Comparison Based on ML Software Framework.

Adapting the original CNN model to TensorFlow Lite and TensorRT formats for deployment on edge devices necessitates an evaluation of the potential impact on model accuracy and efficiency. Converting the model to TensorFlow Lite (TF Lite) and TensorRT incorporates techniques such as post-training quantization and model pruning, which are essential steps to optimize the model for deployment on embedded systems [10]. For example, TensorRT optimizes the original TF model by scanning the graphs and optimizing the subgraphs [61]. After the optimizations by TensorRT, the resulting model can only run on NVIDIA GPUs. TensorFlow Lite models are generated using the TF Lite converter tool, which takes in the standard model and performs specific quantization optimizations and pruning to generate a TFLite model file (.tflite) [62]. This model is run on a CPU and can leverage GPU delegates to speed up executions. Both frameworks leverage the data conversion from a higher precision variable (such as float32) to a smaller precision format (such as int8), which modifies the weights of the model and can result in a model size that is up to 4× smaller than the original [10]. These modifications, while instrumental in reducing the computational and memory footprint of the model, may lead to a slight decrease in model accuracy [63]. As detailed in Figure 9, we conducted a 10-fold cross-validation to compare the performance of the embedded models against the original TensorFlow model, as depicted in Table 4. The comparative analysis reveals a slight decrease in accuracy and an increase in the F1-scores following the conversion to the embedded formats. This suggests that the translation to TensorFlow Lite and TensorRT did not significantly compromise and may, in some aspects, enhance the model’s performance. In terms of model size, the conversion to TensorFlow Lite resulted in a 12.4% reduction, whereas the TensorRT model saw a 12.8% decrease in size.

**Figure 10:**
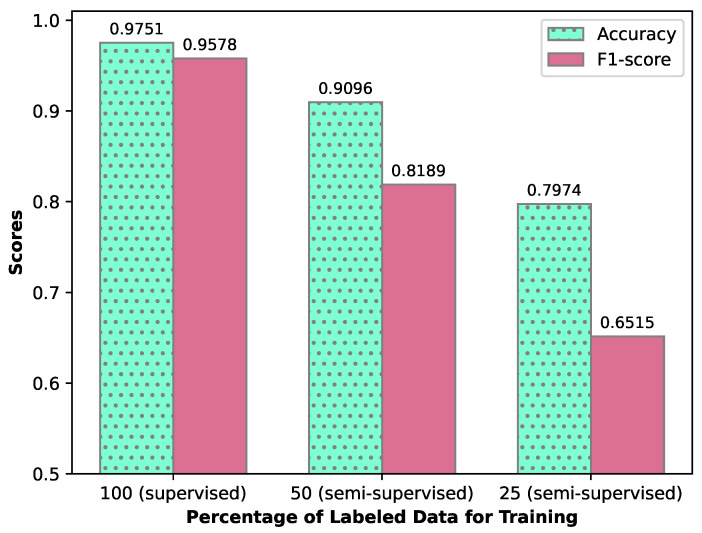
Accuracy and F1-Score as % of labeled data fed to the supervised discriminator in SGAN changes.

In our exploration of a semi-supervised learning approach, we evaluated our CNN model within the semi-supervised generative adversarial network (SGAN) framework, adjusting the ratios of labeled to unlabeled (synthesized) data. The outcomes, including F1 scores and accuracy for varying data proportions, are systematically displayed in Figure 10. Initially, we benchmarked the performance with our fully supervised CNN model. Subsequent trials reveal a decrement in both F1 scores and accuracy upon introducing a 50% blend of labeled and generated data, with a further decline observed when the labeled data is limited to 25%. These observations align with theoretical expectations. Presently, within each training epoch, the discriminator’s weights undergo dual training phases—separately with real and synthesized images—whereas the generator’s weights are adjusted once. This discrepancy suggests an avenue for future exploration; enhancing the SGAN model’s performance might be achievable by equalizing the training frequency of the generator. Additionally, the application of hyper-parameter optimization strategies, such as Bayesian optimization, holds promise for refining SGAN parameters. Data augmentation techniques, including temporal and speed perturbation, are also proposed as methods to improve the model’s semi-supervised learning efficacy, potentially leading to significant improvements in SGAN performance.

### 4.2. Web Platform

**Figure 11:**
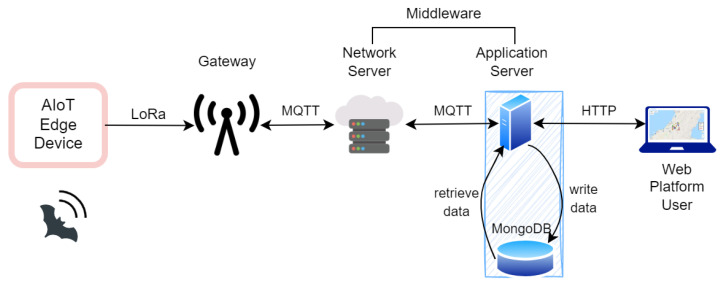
The figure illustrates the system’s network architecture and data flow following the detection of a bat event. The middleware components, specifically the network server (NS) and application server (AS), are responsible for facilitating data collection, storage, and retrieval from edge devices. The NS operates on the publicly available The Things Network (TTN), as depicted in Figure 2. The AS, meanwhile, serves as the foundation of the web platform, enabling user access to stored data through the HTTP protocol and managing interactions with the database for new detection updates or data retrieval requests from web platform users.

In this study, we have developed a comprehensive end-to-end program utilizing our supervised CNN model for real-time bat species detection. Presently, the system features a web-based application interface designed to facilitate interactive user engagement with the detection data via signals received from edge devices. The resulting system, which connects the edge node to the web platform, is highlighted in Figure 11. The next sections will delve into the specifics of the web application component, detailing its implementation and operational functionalities.

#### 4.2.1. Database Management System

We have elected to utilize the document-oriented MongoDB NoSQL database system [64] for data management purposes. Interfacing with the database within the application server is facilitated through the use of the Mongoose library. This setup allows for efficient storage and retrieval of detection data, which is categorized according to three primary attributes: the geographic location of the detection (latitude and longitude coordinates), the timestamp of the detection (formatted according to the ISO 8601-1:2019 standard), and the scientific name of the detected bat species.

#### 4.2.2. Map Interface of Bat Detections

**Figure 12:**
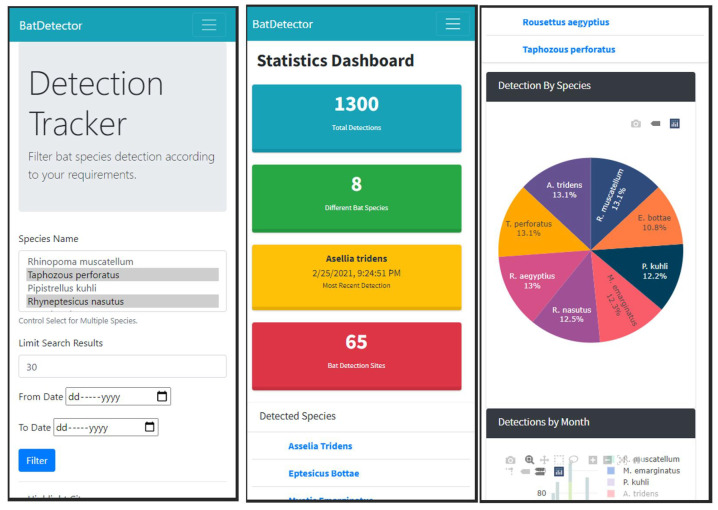
Web app displayed on iPhone X.

The development of the website’s front-end was accomplished using a combination of JavaScript (ES12), HTML 5.0, CSS 3, and Bootstrap 4 [65], ensuring a design that is both responsive and mobile-friendly, as depicted in Figure 12. The backend features an ExpressJS-based web server [66].

**Figure 13:**
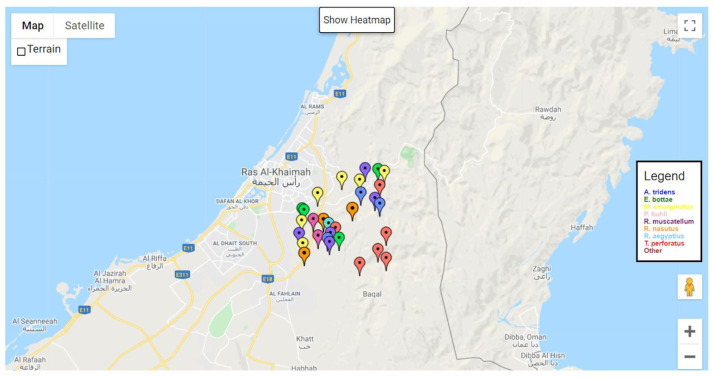
Map interface with bat detection markers.

The web platform’s homepage is characterized by a map interface facilitated through the Google Maps API, which displays markers for each detection event. These markers are accompanied by a color-coded legend to aid in data visualization, as illustrated in Figure 13. Users have the capability to interact with the map, selecting markers to obtain detailed information on specific detection events. Additionally, the interface offers a toggle feature, allowing users to switch between the marker view and a heatmap representation. This heatmap functionality enables an effective visualization of bat population density across different regions, enhancing the user’s ability to interpret and analyze detection data.

Additionally, the web platform offers users the functionality to filter detections based on the species of bat and the date of detection, enhancing the specificity of the data displayed. Users can also limit the number of detections shown on the interface. For those preferring a different data presentation format, an option is provided to display the detections in tabular form. The average response time for the tested web pages is 827 milliseconds, ensuring smooth transitions and accessibility for the end user.

#### 4.2.3. Dashboard Interface of Bat Detections

The dashboard section of the web application is designed to present a comprehensive summary of the bat detections, aggregating data stored within the database. To develop this interactive dashboard, we employed the Plotly JavaScript library, chosen for its robust feature set that includes versatile filter controls integrated with the charts. These controls enable users to interactively customize their data visualization experience, such as by selecting specific time ranges for line charts or choosing to include or exclude particular bat species from the displayed chart. Further visualizations of our front-end interface are provided in the GitHub repository.

### 4.3. Edge Device Analysis

**Figure 14:**
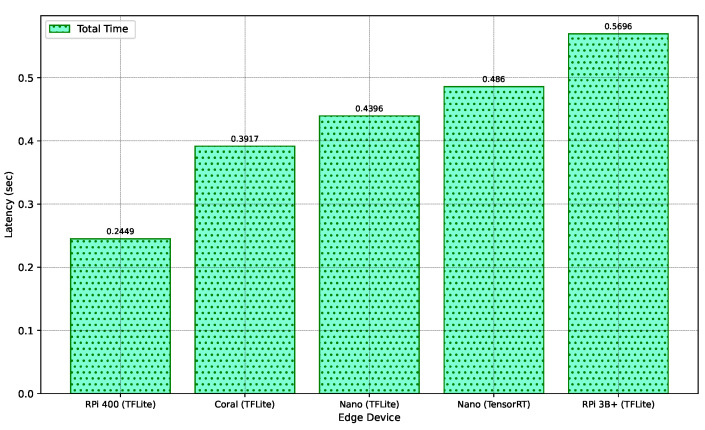
Latency by Edge Device.

In our study, the selection of edge devices for deploying our model was rigorously assessed based on key performance metrics: latency, power consumption (measured as current drawn), and CPU utilization. Latency measurements, cataloged in ascending order, are depicted in Figure 14, captured within a 10-min interval. The figure elucidates the aggregate duration, encompassing both preprocessing and CNN model inference times. Notably, the compact nature of our model results in the inference phase constituting less than 10% of the total processing time. Given the processing of 3-s audio segments for spectrogram analysis, all evaluated edge devices were deemed compatible with our requirements. By analyzing the audio segment and transmitting the signal through the LoRaWAN protocol, real-time classification of subsequent audio segments in the buffer is facilitated. Particularly, the TensorFlow Lite modified model, when executed on an RPi 400, demonstrated the most efficient performance, achieving the lowest average latency of 0.39 s. Conversely, the RPi 3B+ exhibited a marginally higher latency, recording a total time of 0.57 s while running the identical model. The Jetson Nano, despite its TensorRT optimization, exhibited a longer total processing time. This observation suggests that, contrary to typical expectations where GPU and TPU-equipped microcomputers excel in machine learning tasks, our model’s compactness and efficiency do not significantly leverage advanced computational resources. For devices further constrained to computational resources, savings can be introduced in the audio-to-spectrogram conversion process. One could opt to use the STFT-based spectrogram, which does not require the additional mel-filters to be applied to generate the MSFB. Alternatively, a more practical approach can involve using algorithms that can reduce the overall computations to generate the spectrogram, such as nnAudio, which can be four times faster than the librosa package in producing spectrograms for the same input signal [67].

**Figure 15:**
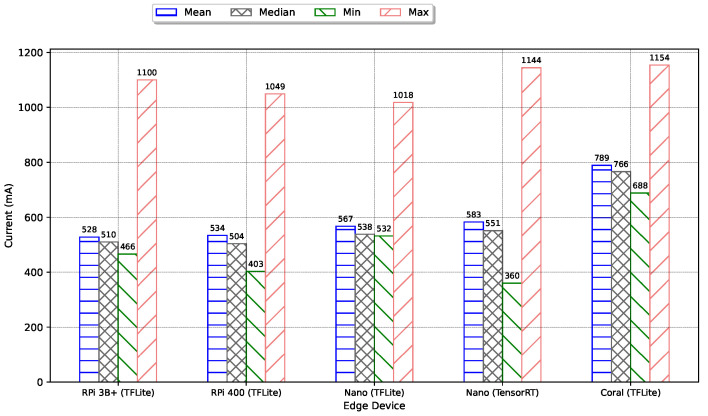
Current drawn by Edge Device.

We also test the current drawn by each of the edge devices for a fixed voltage source of 5 V. The summary of the current drawn by each device over a 10-min window is presented in Figure 15. The current was measured using a digital USB ammeter (Yocto-Amp [68]) connected in series with the edge devices. The measured values in mA are then read via a USB serial interface. The CPU-based RPi 3B+, followed by the RPi 400, consumes the least amount of current, making the RPi 3B+ most suitable for deployment in a power-constrained remote setting. The Google Coral running the TPU consumed the most current, with a mean of 789 mA. A Kruskal-Wallis test shows that there is a statistically significant difference in current consumption between the 5 test cases with a *p*-value of 0.0. All the maximum currents drawn are compared against the hardware datasheet, and they are lower than the recommended maximum.

In a battery-powered configuration utilizing the RPi 3B+ with an average current draw of 528 mA from a 5 V source, the operational power demand of the system is approximately 2.64 W. Employing a 50 Ah commercial power bank as the energy reserve, the effective output is calculated to be 250 Wh (50 Ah × 5 V), projecting the operational longevity of our edge device on this power supply to be around 94 h (250/2.64). Considering the variable nature of CPU usage, a conservative estimate adjusts the expected battery life to 50% of this duration [69], translating to an operational range between 47 and 94 h without the need for manual intervention. Previous work [70] has shown that integrating an RPi with a 12 V 50 W solar panel with a solar charger and a 12 V battery can facilitate continuous operation in the field, devoid of frequent maintenance needs.

**Figure 16:**
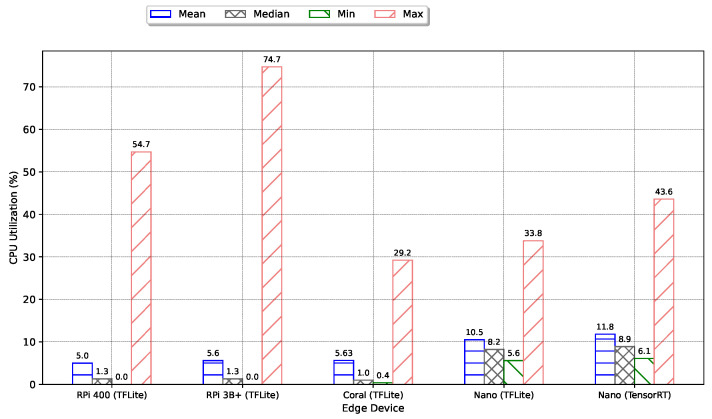
CPU Utilization by Edge Device.

**Figure 17:**
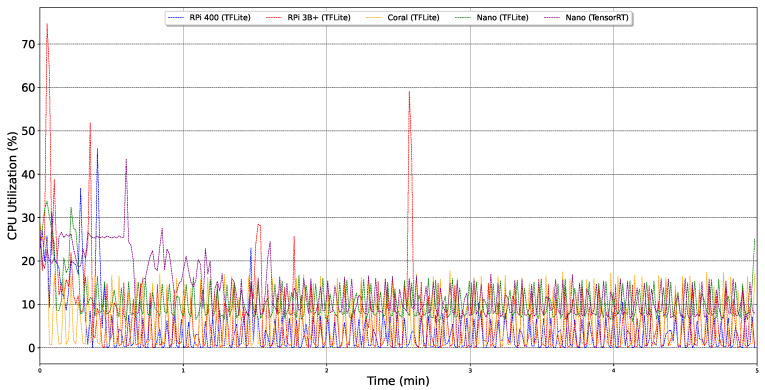
Graph of CPU utilization over 5 min per edge device.

Figure 16 shows the CPU utilization percentages for various edge devices, revealing that the average utilization across all devices does not exceed 12%, with the RPi 3B+ peaking at under 75% utilization. Additionally, temperature measurements for these devices confirmed adherence to operational norms set forth in their respective datasheets. A detailed analysis of CPU utilization, captured over a five-minute interval while the CNN model operates on these devices, is illustrated in Figure 17. Initial fluctuations observed across all devices can be attributed to the operating system’s memory allocation processes and the setup phase. Notably, when running the TensorRT model, the Jetson Nano exhibits more pronounced variations in CPU utilization compared to the TensorFlow Lite (TFLite) model, offering insights into its differential impact on power usage. The RPi 3B+ experiences a significant utilization spike at the simulation’s onset and another at approximately the halfway mark. Despite its lower overall power consumption, selecting the RPi 3B+ necessitates consideration of these initial power surges.

A detailed breakdown of the system’s total cost, amounting to $712.75 at the time of purchase, is provided for reference. The RPi 3B+ is selected for this analysis due to its smaller current requirements as well as its most affordable cost. This cost encompasses various components: the LoRa Raspberry Pi Gateway with Enclosure ($199.95), the SparkFun LoRa Gateway—1-Channel (ESP32) ($34.95), the Pettersson M500-384 USB Ultrasound Microphone ($342.00), the Adafruit 6 V 6 W Solar Panel ($69.00), a Solar Lithium Ion/Polymer charger ($17.50), a Lithium Ion Polymer Battery ($9.95), and the Raspberry Pi 3B+ ($40.00) [63]. This cost analysis offers a comprehensive view of the financial requirements for implementing such a system. In comparison, the SonoBat 4 (version 3.1.7p) software suite itself costs $680 for the Universal package and $1536 for the North American version [7], while the SM4BAT bat recorder system by Kaleidoscope costs $999 [8].

## 5. Conclusions

This study presents a supervised algorithm pipeline equipped for edge-based bat species classification, utilizing a compact CNN model architecture. The proposed model can accurately classify eight bat species with an accuracy of 97.5% and an F1-score of 0.9578. This resulted in an integrated end-to-end system that efficiently transmits detection data from edge devices to a backend server via the LoRaWAN protocol and The Things Network. To enhance user interaction and data accessibility, we designed a responsive website that showcases real-time bat detections. This platform features both a map view for geographical tracking and a dashboard summary for aggregated insights. Drawing on previous findings that underscore the potential for technology to reduce errors and streamline data collection [71], our framework promises to enrich our comprehension of bat species and contribute to ecosystem health assessments. We also explore the data-constraint training of our proposed CNN model in a semi-supervised SGAN framework to perform exploratory analysis on its robustness when labeled data is limited. By leveraging a generator network, the model is still able to perform relatively well within a smaller percentage of labeled data. Additionally, in a field where open-source resources are notably scarce [6], our contribution of openly available code stands to catalyze progress in bioacoustic research, offering a valuable starting point for projects embarking on machine learning and software development in this domain.

One of the limitations inherent in our present study concerns the absence of generalizability to other bat species. Given that our dataset comprises 8 bat species frequently encountered in the UAE, it will be necessary to do further retraining and parameter tweaking for species not examined in this project. Furthermore, it should be noted that our system has not been subjected to site testing in order to evaluate the potential effects of the environment and wildlife on the field deployment. Potential future endeavors may involve extending our research to develop a comprehensive feature extractor for bat species that can optimize various AI tasks. In future research, we also aim to investigate other semi-supervised studies further to assess more effective frameworks for training models with limited data. Finally, further investigation is necessary to examine the bat behavior that is inherent in the bat echolocation calls through the application of unsupervised learning techniques.

## Figures and Tables

**Table 1:** Specifications of the Pettersson M500-384 [54] Ultrasound Microphone. The device is sourced from Pettersson Elektronik AB, Uppsala, Sweden.

Feature	Specification
Weight	60 g
Dimensions	43 × 114 × 13 mm
Sampling Frequency	384 kHz
ADC Resolution	16 bits
Frequency Range	10–160 kHz
Compatibility	Computers/laptops/tablets/smartphones with Android, Linux (Ubuntu), OS X, iOS, or Windows
Drivers	Standard sound device drivers
Enclosure	Slim and durable aluminum
Microphone Technology	Advanced electret (similar to D500X)
Modes	Directional and omni-directional
Software Compatibility	Must support recording at 384 kHz. Compatible with BatSound and BatSound Touch (Windows), Audacity (Windows, macOS, Linux)
Interface	USB 2.0, full speed, OTG/host
Anti-aliasing Filter	8th order, 160 kHz
Power	USB bus powered

**Table 2:** Specifications of the LoRaWAN Adapter.

Attribute	Details
Modem	Hope RFM95W
Frequency Range	868/915 MHz
Ports	CH340C USB-to-Serial interface & Micro-B USB connector for power and programming
Power	350 mW (5 V @ 70 mA)

**Table 3:** Specifications of the LoRaWAN Gateway. The device is sourced from SparkFun Electronics, Colorado, USA.

Attribute	Details
Engine	LoRa Concentrator Engine: Semtech SX1301
Sensitivity	upto −142.5 dBm (Spreading Factor 12, Bandwidth 125 KHz)
Range	15 km (Line of Sight), 2 km in dense urban areas
Temperature	−40 to 85 °C
Power Output	350 mW/25.5 dBm
Antennae	2x SX1257 as Tx/Rx front-ends

**Table 4:** Architecture of the proposed compact CNN model. The Total parameters amount to 204,256 (797.88 KB), and the Trainable parameters amount to 203,648 (795.50 KB).

Layer (Type)	Output Shape	Param #
InputLayer	(None, 112, 170, 3)	0
Conv2D	(None, 56, 85, 56)	8288
BatchNormalization	(None, 56, 85, 56)	224
Activation (ReLU)	(None, 56, 85, 56)	0
MaxPooling2D	(None, 28, 43, 56)	0
Dropout	(None, 28, 43, 56)	0
Conv2D	(None, 14, 22, 72)	100,872
BatchNormalization	(None, 14, 22, 72)	288
Activation (ReLU)	(None, 14, 22, 72)	0
AveragePooling2D	(None, 7, 11, 72)	0
Dropout	(None, 7, 11, 72)	0
Conv2D	(None, 7, 11, 56)	36,344
BatchNormalization	(None, 7, 11, 56)	224
Activation (ReLU)	(None, 7, 11, 56)	0
AveragePooling2D	(None, 4, 6, 56)	0
Dropout	(None, 4, 6, 56)	0
Conv2D	(None, 4, 6, 72)	36,360
BatchNormalization	(None, 4, 6, 72)	288
Activation (ReLU)	(None, 4, 6, 72)	0
AveragePooling2D	(None, 2, 3, 72)	0
Dropout	(None, 2, 3, 72)	0
Flatten	(None, 432)	0
Dense	(None, 48)	20,784
BatchNormalization	(None, 48)	192
Activation (ReLU)	(None, 48)	0
Dropout	(None, 48)	0
Output (Softmax)	(None, 8)	392

**Table 5:** Samples per Strata with the Stratified Sampling Strategy.

Species	Training Samples	Validation Samples	Testing Samples
*R. muscatellum*	1199	300	167
*T. perforatus*	290	73	40
*P. kuhli*	215	54	30
*R. nasutus*	194	48	27
*E. bottae*	89	22	12
*R. aegyptius*	87	22	12
*M. emarginatus*	81	20	11
*A. tridens*	18	5	3

**Table 6:** Architecture of the Generator model in SGAN. The input shape of the discriminator model was modified to allow the synthetic image produced by the generator model to be used in the training.

Layer (Type)	Output Shape	Param #
InputLayer	(None, 100)	0
Dense	(None, [150,528])	15,203,328
LeakyReLU	(None, [150,528])	0
Reshape	(None, 28, 42, 128)	0
Conv2DTranspose	(None, 56, 84, 128)	262,272
LeakyReLU	(None, 56, 84, 128)	0
Conv2DTranspose	(None, 112, 168, 128)	262,272
LeakyReLU	(None, 112, 168, 128)	0
Conv2D	(None, 112, 168, 3)	18,819

## Data Availability

The dataset analyzed in this study is restricted by Emirates Nature—WWF. The complete source code of our experiment is publicly available on https://github.com/Taslim-M/Bat2Web (accessed on 5 February 2024).

## Abbreviations

The following abbreviations are used in this manuscript:

CNN	Convolutional neural network
ML	Machine learning
IoT	Internet of Things
MSFB	Mel-scaled Filter Bank
NN	Neural Network
STFT	Short-time Fourier Transform

## References

[1] Browning E., Gibb R., Glover-Kapfer P., Jones K.E. (2017). Passive Acoustic Monitoring in Ecology and Conservation.

[2] Russ J. (2012). British Bat Calls: A Guide to Species Identification.

[3] Adams A.M., Jantzen M.K., Hamilton R.M., Fenton M.B. (2012). Do you hear what I hear? Implications of detector selection for acoustic monitoring of bats. Methods Ecol. Evol..

[4] Mac Aodha O., Gibb R., Barlow K.E., Browning E., Firman M., Freeman R., Harder B., Kinsey L., Mead G.R., Newson S.E. (2018). Bat detective—Deep learning tools for bat acoustic signal detection. PLoS Comput. Biol..

[5] Emirates Nature-WWF. https://www.emiratesnaturewwf.ae/en.

[6] Alipek S., Maelzer M., Paumen Y., Schauer-Weisshahn H., Moll J. (2023). An Efficient Neural Network Design Incorporating Autoencoders for the Classification of Bat Echolocation Sounds. Animals.

[7] Sonobat Software. https://sonobat.com/.

[8] Kaleidoscope Pro Analysis Software. https://www.wildlifeacoustics.com/products/kaleidoscope-pro.

[9] Hamdan S., Ayyash M., Almajali S. (2020). Edge-computing architectures for internet of things applications: A survey. Sensors.

[10] Merenda M., Porcaro C., Iero D. (2020). Edge machine learning for ai-enabled iot devices: A review. Sensors.

[11] Cruz M., Mafra S., Teixeira E., Figueiredo F. (2022). Smart strawberry farming using edge computing and IoT. Sensors.

[12] Premsankar G., Di Francesco M., Taleb T. (2018). Edge computing for the Internet of Things: A case study. IEEE Internet Things J..

[13] Chen B., Wan J., Celesti A., Li D., Abbas H., Zhang Q. (2018). Edge computing in IoT-based manufacturing. IEEE Commun. Mag..

[14] Reuther A., Michaleas P., Jones M., Gadepally V., Samsi S., Kepner J. Survey and benchmarking of machine learning accelerators. Proceedings of the 2019 IEEE high performance extreme computing conference (HPEC).

[15] Jetson Nano Developer Kit. https://developer.nvidia.com/embedded/jetson-nano-developer-kit.

[16] Dev Board|Coral. https://coral.ai/products/dev-board.

[17] Antonini M., Vu T.H., Min C., Montanari A., Mathur A., Kawsar F. Resource characterisation of personal-scale sensing models on edge accelerators. Proceedings of the First International Workshop on Challenges in Artificial Intelligence and Machine Learning for Internet of Things.

[18] Silveira O.C., de Melo J.G., Moreira L.A., Pinto J.B., Rodrigues L.R., Rosa P.F. Evaluating a visual simultaneous localization and mapping solution on embedded platforms. Proceedings of the 2020 IEEE 29th International Symposium on Industrial Electronics (ISIE).

[19] Ikpehai A., Adebisi B., Rabie K.M., Anoh K., Ande R.E., Hammoudeh M., Gacanin H., Mbanaso U.M. (2018). Low-power wide area network technologies for Internet-of-Things: A comparative review. IEEE Internet Things J..

[20] Ayoub W., Samhat A.E., Nouvel F., Mroue M., Prévotet J.C. (2018). Internet of mobile things: Overview of lorawan, dash7, and nb-iot in lpwans standards and supported mobility. IEEE Commun. Surv. Tutor..

[21] Ballerini M., Polonelli T., Brunelli D., Magno M., Benini L. Experimental evaluation on NB-IoT and LoRaWAN for industrial and IoT applications. Proceedings of the 2019 IEEE 17th International Conference on Industrial Informatics (INDIN).

[22] Lalle Y., Fourati L.C., Fourati M., Barraca J.P. A comparative study of lorawan, sigfox, and nb-iot for smart water grid. Proceedings of the 2019 Global Information Infrastructure and Networking Symposium (GIIS).

[23] El Chall R., Lahoud S., El Helou M. (2019). LoRaWAN network: Radio propagation models and performance evaluation in various environments in Lebanon. IEEE Internet Things J..

[24] Fraga-Lamas P., Celaya-Echarri M., Azpilicueta L., Lopez-Iturri P., Falcone F., Fernández-Caramés T.M. (2019). Design and empirical validation of a LoRaWAN IoT smart irrigation system. Proceedings.

[25] Codeluppi G., Cilfone A., Davoli L., Ferrari G. (2020). LoRaFarM: A LoRaWAN-based smart farming modular IoT architecture. Sensors.

[26] Ahmed M.A., Gallardo J.L., Zuniga M.D., Pedraza M.A., Carvajal G., Jara N., Carvajal R. (2022). LoRa based IoT platform for remote monitoring of large-scale agriculture farms in Chile. Sensors.

[27] Ayele E.D., Meratnia N., Havinga P.J. An asynchronous dual radio opportunistic beacon network protocol for wildlife monitoring system. Proceedings of the 2019 10th IFIP International Conference on New Technologies, Mobility and Security (NTMS).

[28] Turchet L., Fazekas G., Lagrange M., Ghadikolaei H.S., Fischione C. (2020). The internet of audio things: State of the art, vision, and challenges. IEEE Internet Things J..

[29] Blumstein D.T., Mennill D.J., Clemins P., Girod L., Yao K., Patricelli G., Deppe J.L., Krakauer A.H., Clark C., Cortopassi K.A. (2011). Acoustic monitoring in terrestrial environments using microphone arrays: Applications, technological considerations and prospectus. J. Appl. Ecol..

[30] Rhinehart T.A., Chronister L.M., Devlin T., Kitzes J. (2020). Acoustic localization of terrestrial wildlife: Current practices and future opportunities. Ecol. Evol..

[31] Purwins H., Li B., Virtanen T., Schlüter J., Chang S.Y., Sainath T. (2019). Deep learning for audio signal processing. IEEE J. Sel. Top. Signal Process..

[32] Kutsumi Y., Kanegawa N., Zeida M., Matsubara H., Murayama N. (2022). Automated bowel sound and Motility analysis with CNN using a smartphone. Sensors.

[33] Peruzzi G., Galli A., Pozzebon A. A novel methodology to remotely and early diagnose sleep bruxism by leveraging on audio signals and embedded machine learning. Proceedings of the 2022 IEEE International Symposium on Measurements & Networking (M&N).

[34] Tariq Z., Shah S.K., Lee Y. (2022). Feature-based fusion using CNN for lung and heart sound classification. Sensors.

[35] Henry F., Parsi A., Glavin M., Jones E. (2023). Experimental Investigation of Acoustic Features to Optimize Intelligibility in Cochlear Implants. Sensors.

[36] Di Maggio L.G. (2022). Intelligent fault diagnosis of industrial bearings using transfer learning and CNNs pre-trained for audio classification. Sensors.

[37] Jung H., Choi S., Lee B. (2023). Rotor fault diagnosis method using CNN-Based transfer learning with 2D sound spectrogram analysis. Electronics.

[38] Tran T., Lundgren J. (2020). Drill fault diagnosis based on the scalogram and mel spectrogram of sound signals using artificial intelligence. IEEE Access.

[39] Bergler C., Schröter H., Cheng R.X., Barth V., Weber M., Nöth E., Hofer H., Maier A. (2019). ORCA-SPOT: An automatic killer whale sound detection toolkit using deep learning. Sci. Rep..

[40] Li P., Liu X., Palmer K., Fleishman E., Gillespie D., Nosal E.M., Shiu Y., Klinck H., Cholewiak D., Helble T. Learning deep models from synthetic data for extracting dolphin whistle contours. Proceedings of the 2020 International Joint Conference on Neural Networks (IJCNN).

[41] Shang Y., Li J. Study on echo features and classification methods of fish species. Proceedings of the 2018 10th International Conference on Wireless Communications and Signal Processing (WCSP).

[42] Ruff Z.J., Lesmeister D.B., Duchac L.S., Padmaraju B.K., Sullivan C.M. (2020). Automated identification of avian vocalizations with deep convolutional neural networks. Remote Sens. Ecol. Conserv..

[43] Kojima R., Sugiyama O., Hoshiba K., Suzuki R., Nakadai K. HARK-Bird-Box: A portable real-time bird song scene analysis system. Proceedings of the 2018 IEEE/RSJ International Conference on Intelligent Robots and Systems (IROS).

[44] Ko K., Park S., Ko H. Convolutional feature vectors and support vector machine for animal sound classification. Proceedings of the 2018 40th Annual International Conference of the IEEE Engineering in Medicine and Biology Society (EMBC).

[45] Lostanlen V., Salamon J., Farnsworth A., Kelling S., Bello J.P. (2019). Robust sound event detection in bioacoustic sensor networks. PLoS ONE.

[46] Lin T.H., Yang H.T., Huang J.M., Yao C.J., Lien Y.S., Wang P.J., Hu F.Y. Evaluating changes in the marine soundscape of an offshore wind farm via the machine learning-based source separation. Proceedings of the 2019 IEEE Underwater Technology (UT).

[47] Potamitis I. (2015). Unsupervised dictionary extraction of bird vocalisations and new tools on assessing and visualising bird activity. Ecol. Inform..

[48] Hughes A.C., Satasook C., Bates P.J., Soisook P., Sritongchuay T., Jones G., Bumrungsri S. (2011). Using echolocation calls to identify Thai bat species: Vespertilionidae, Emballonuridae, Nycteridae and Megadermatidae. Acta Chiropterol..

[49] Skalak S.L., Sherwin R.E., Brigham R.M. (2012). Sampling period, size and duration influence measures of bat species richness from acoustic surveys. Methods Ecol. Evol..

[50] Fukui D., Hill D.A., Kim S.S., Han S.H. (2015). Echolocation call structure of fourteen bat species in Korea. Anim. Syst. Evol. Divers..

[51] Tabak M.A., Murray K.L., Reed A.M., Lombardi J.A., Bay K.J. (2022). Automated classification of bat echolocation call recordings with artificial intelligence. Ecol. Inform..

[52] Paumen Y., Mälzer M., Alipek S., Moll J., Lüdtke B., Schauer-Weisshahn H. (2022). Development and test of a bat calls detection and classification method based on convolutional neural networks. Bioacoustics.

[53] Yoh N., Kingston T., McArthur E., Aylen O.E., Huang J.C.C., Jinggong E.R., Khan F.A.A., Lee B.P., Mitchell S.L., Bicknell J.E. (2022). A machine learning framework to classify Southeast Asian echolocating bats. Ecol. Indic..

[54] Pettersson Elektronik A.B. M500-384 USB Ultrasound Microphone. https://batsound.com/product/m500-384-usb-ultrasound-microphone/.

[55] Ghimire D., Kil D., Kim S.h. (2022). A survey on efficient convolutional neural networks and hardware acceleration. Electronics.

[56] Zualkernan I., Judas J., Mahbub T., Bhagwagar A., Chand P. A tiny CNN architecture for identifying bat species from echolocation calls. Proceedings of the 2020 IEEE/ITU International Conference on Artificial Intelligence for Good (AI4G).

[57] Goel S., Pangasa R., Dawn S., Arora A. Audio acoustic features based tagging and comparative analysis of its classifications. Proceedings of the 2018 Eleventh International Conference on Contemporary Computing (IC3).

[58] Li L., Jamieson K., DeSalvo G., Rostamizadeh A., Talwalkar A. (2018). Hyperband: A novel bandit-based approach to hyperparameter optimization. J. Mach. Learn. Res..

[59] Andrade G., Rodrigues M., Novais P. (2022). A Survey on the Semi Supervised Learning Paradigm in the Context of Speech Emotion Recognition. Intelligent Systems and Applications: Proceedings of the 2021 Intelligent Systems Conference (IntelliSys), Amsterdam, The Netherlands, 2–3 September 2021.

[60] Khan N., Sarkar N. (2022). Semi-supervised generative adversarial network for stress detection using partially labeled physiological data. arXiv.

[61] Verma G., Gupta Y., Malik A.M., Chapman B. Performance evaluation of deep learning compilers for edge inference. Proceedings of the 2021 IEEE International Parallel and Distributed Processing Symposium Workshops (IPDPSW).

[62] Warden P., Situnayake D. (2019). Tinyml: Machine Learning with Tensorflow Lite on Arduino and Ultra-Low-Power Microcontrollers.

[63] Zualkernan I., Judas J., Mahbub T., Bhagwagar A., Chand P. An aiot system for bat species classification. Proceedings of the 2020 IEEE International Conference on Internet of Things and Intelligence System (IoTaIS).

[64] Gyorödi C.A., Dumşe-Burescu D.V., Zmaranda D.R., Gyorödi R.Ş. (2022). A comparative study of MongoDB and document-based MySQL for big data application data management. Big Data Cogn. Comput..

[65] Krause J. (2020). Introducing Bootstrap 4: Create Powerful web Applications Using Bootstrap 4.5.

[66] Sharma M. (2022). Full Stack Development with MongoDB: Covers Backend, Frontend, APIs, and Mobile App Development Using PHP, NodeJS, ExpressJS, Python and React Native.

[67] Cheuk K.W., Anderson H., Agres K., Herremans D. (2020). nnaudio: An on-the-fly gpu audio to spectrogram conversion toolbox using 1d convolutional neural networks. IEEE Access.

[68] Yoctopuce. Yocto-Amp: USB Amperometric Sensor. https://www.yoctopuce.com/EN/products/usb-electrical-sensors/yocto-amp.

[69] Proppe D.S., Pandit M.M., Bridge E.S., Jasperse P., Holwerda C. (2020). Semi-portable solar power to facilitate continuous operation of technology in the field. Methods Ecol. Evol..

[70] Jolles J.W. (2021). Broad-scale applications of the Raspberry Pi: A review and guide for biologists. Methods Ecol. Evol..

[71] Olson D.D., Bissonette J.A., Cramer P.C., Green A.D., Davis S.T., Jackson P.J., Coster D.C. (2014). Monitoring wildlife-vehicle collisions in the information age: How smartphones can improve data collection. PLoS ONE.

